# F109. BOUNDARIES BETWEEN DEFICIT AND NONDEFICIT SCHIZOPHRENIA: LONG TERM STABILITY AND OUTCOMES

**DOI:** 10.1093/schbul/sby017.640

**Published:** 2018-04-01

**Authors:** Gustavo Mustafé, Diego Mendes, Thaís Martins, Thalita Fernandes, Clarissa Dantas

**Affiliations:** Medical School, State University of Campinas

## Abstract

**Background:**

Negative symptoms of schizophrenia are admittedly associated with a poorer outcome regarding aspects such as functionality, quality of life and cognitive performance. Patients with prominent, persistent and primary negative symptoms have been considered to manifest a putative subtype called “Deficit schizophrenia” (DS). However, the boundaries of deficit and nondeficit forms were put in question since the publication of a study that considered separately a group of patients with persistent negative symptoms whose primary nature could not be asserted, the “ambiguous nondeficit” group, who would be otherwise categorized as nondefict according to the gold standard instrument: the Schedule for the Assessment of the Deficit Syndrome (SDS). Those patients presented psychopathological features, quality of life, insight and cognitive function quite different from the nondeficit group, and closer to the deficit group. The objectives of the present study are to investigate the stability of the categorization regarding the presence of DS in three groups: “deficit”(DS), “nondeficit” (ND) and “ambiguous nondeficit” (SND) over a long term follow-up and to evaluate clinical outcomes in the different groups.

**Methods:**

We will contact 85 patients with schizophrenia, considered clinically stable in the previous year, who participated in a study about the DS in 2009/2010. Back then, they were recruited in two sites: an outpatient service of a university general hospital (49 patients) and a community-based mental health service (36 patients). Patients will be assessed with the same instruments adopted in the first study: a questionnaire for clinical and demographic information; BPRS, SAPS, SANS, Calgary Depression Scale, the SDS, QLS, and a battery of neurocognitive tests. We started the recruitment by the patients originally treated in the outpatient clinic.

**Results:**

Here we present partial results. Of the 49 patients, 5 refused to participate in the follow-up study, 3 died prematurely, and 1 had the diagnostic changed for bipolar disorder. Assessment interval was 6.9 years ± 0.5 Among the 20 reassessed patients, mean age at baseline was 36.9 ± 8.9 years, mean duration of mental illness was 16 ± 10.1 years, and 75% were men. They had in mean, 10.7 ± 3.3 years of education, only 20% had any work activity, 15% were married and 55% had a low socioeconomic position. These demographic aspects slightly worsened: only 15% had an occupation at follow-up, and 60% fell in the lower socioeconomic position. Regarding the SDS classification, 4 of 9 ND patients at the baseline were reclassified as DS; 1 of 7 DS was reclassified as ND, the other 6 remained DS; from the AND, 3 were considered DS and 1 ND, from a total of 4. At the end, there were 13 DS and 7 ND, while at the baseline they were: 7 DS, 9 ND and 4 AND. Concerning psychopathology, 80% of the patients had an increase in SANS and the most expressive increase was in nondeficit group (an average of 5.4 points), although the average in DS group remained the higher (18.9 points). Still, SAPS and Calgary remained low in all three subgroups, with a mean of 6.20 and 2.20 points, respectively. As to medication, 70% of the baseline were in use of Clozapine (67% of ND, 57% of DS and 100% of the AND group) and that total number remained the same during the follow up.

**Discussion:**

Our preliminary results are derived from a small sample. Although we cannot draw definite conclusion, these outcomes suggest trends that are worth observing: the worsening of negative symptoms among patients and the tendency of conversion to DS group, especially among the “ambiguous” group. This advocates against the dichotomous division of deficit and nondeficit schizophrenia and speaks in favor of a dimensional understanding of negative symptoms.

